# Pilot study with IBAT inhibitor A4250 for the treatment of cholestatic pruritus in primary biliary cholangitis

**DOI:** 10.1038/s41598-018-25214-0

**Published:** 2018-04-27

**Authors:** Samer Al-Dury, Annika Wahlström, Staffan Wahlin, Jacqueline Langedijk, Ronald Oude Elferink, Marcus Ståhlman, Hanns-Ulrich Marschall

**Affiliations:** 1 Sahlgrenska Academy, Institute of Medicine, Department of Molecular and Clinical Medicine and Wallenberg Laboratory, University of Gothenburg, Gothenburg, Sweden; 2 Karolinska University Hospital Huddinge, Department of Gastroenterology and Hepatology, Stockholm, Sweden; 3 Tytgat Institute for Liver and Intestinal Research, Department of Gastroenterology and Hepatology, Academic Medical Center, University of Amsterdam, Amsterdam, The Netherlands

## Abstract

Pruritus is a common complication of cholestatic liver diseases. Inhibition of the ileal bile acid transporter (IBAT/ASBT) may emerge as treatment option. Our aim was to assess tolerability and effect on pruritus of the selective IBAT inhibitor A4250 in patients with primary biliary cholangitis (PBC). Ten patients with PBC and bile acid sequestrant treatment of cholestatic pruritus were after a two-week wash out of the bile acid sequestrant treated with either 0.75 mg (n = 4) or 1.5 mg (n = 5) of A4250 for four weeks. Patients’ pruritus was assessed by Visual Analogue Scale (VAS), 5-D itch scale and the pruritus module of the PBC40 questionnaire. Plasma bile acids and 7α-hydroxy-4-cholesten-3-one were measured by UPLC-MS/MS, plasma fibroblast growth factor 19 by ELISA, and serum autotaxin activity by homemade assay. All nine patients exposed to A4250 reported a remarkable improvement in pruritus, until none or mild according to 5-D itch, VAS and PBC40 pruritus. Five patients finished the study prematurely due to abdominal pain (5/5) and diarrhoea (4/5). The high incidence of probably bile acid malabsorption-related diarrhoea and abdominal pain in the bile acid sequestrant pre-treated population indicates that the start dose of A4250 may have been too high for adult patients.

## Introduction

Primary Biliary Cholangitis (PBC) is a chronic immune-mediated liver disease characterized by progressive cholestasis, biliary fibrosis and eventually cirrhosis^1^. Pruritus (itch) is a frequent and troublesome symptom, seen in 60–70% of patients at some point during the disease process^2^. The pathogenesis of cholestatic pruritus is complex and several putative pruritogens have been proposed, including circulating bile acids^3^. The use of ursodeoxycholic acid (UDCA), the standard of care in PBC, has improved outcomes in PBC but has not been shown to improve pruritus^4^. Second-line treatment of PBC with obeticholic acid may even deteriorate pruritus^5^. Bile acid sequestrants such as cholestyramine and colestipol are often administered to treat pruritus, but their effectiveness in practice is limited. Despite its modest evidence, and poor tolerability profile, cholestyramine is the only drug licensed for the treatment of PBC-related pruritus^4^. Of note, a randomized, placebo-controlled trial with the potent bile acid sequestrant colesevelam was unable to demonstrate a better relief from cholestatic pruritus than placebo^6^. Rifampicin as second-line therapy for cholestatic pruritus has a success rate of about 50% in clinical practice but is hampered by hepatotoxic side effects^7^. Other drug therapies including opiate antagonists as third-line therapy, and selective serotonin uptake inhibitors or gababentin are less well documented^4^. Nasobiliary drainage is only a temporary invasive and uncomfortable intervention^8^. Since all available treatment options for cholestatic pruritus lack long-term efficacy and have side effects^9^ liver transplantation may be indicated even without advanced liver failure. For those reasons, there is a high need to find an effective and safe antipruritic treatment for patients with PBC and other cholestatic liver diseases that are complicated by pruritus.

The ileal bile acid transporter (IBAT, SLC10A2), also called apical sodium-dependent bile salt transporter (ASBT), is a key element in the enterohepatic circulation of bile acids. It is an integral brush border membrane glycoprotein mainly expressed in the distal ileum^10^ and responsible for the reabsorption of about 95% of the intestinal bile acids, predominantly in the glycine- or taurine-conjugated form, that are then recirculated to the liver via portal venous blood. Lowering the bile acid pool by IBAT inhibition may emerge as an option for the treatment of cholestatic pruritus.

A4250 is a small compound (molecular weight, 740.9 g/mol) that showed significant improvement of bile acid-associated hepatobiliary injury in MDR2 (ABCB4) knock-out mice, an established animal model of cholestatic liver disease^11^. We have recently shown in a phase I trial that oral administration of A4250 in healthy volunteers induced substantial effects on bile acid synthesis and plasma and faecal bile acids, which likely results from the decreased ileal FXR-dependent FGF19 secretion. Treatment with A4250 was not associated with adverse events other than those associated with the mechanism of action of an IBAT inhibitor, i.e. bile acid-induced increase in the number of bowel movements^12^.

The aim of our current pilot study was to assess safety and tolerability, and potential improvements of pruritus of oral A4250 in patients with PBC and bile acid sequestrant pre-treated cholestatic pruritus.

## Results

### Demographics

A4250PBCpruritus (Clinical Trial registration NCT02360852, dated 1/14/2015) was an open-label exploratory phase IIa study. Patients with PBC fulfilling inclusion criteria were screened from local data bases at Sahlgrenska and Karolinska University Hospitals consisting of about 500 patients of which slightly more than 10% had been prescribed a bile acid sequestrant for cholestatic pruritus. All those patients were on continuous UDCA 10–15 mg/kg/d and classified as non-responders according to Toronto criterion, i.e. ALP >1.67 ULN for more than one year^13^. UDCA was continued at the same dose throughout the study. A total of ten patients, nine females, one male, 54.9 ± 14.3 years of age were included, eight of them treated with cholestyramine 4–8 g/day and two of them with colestipol 5–10 g/day, which were the highest individually tolerated doses.

### Safety and tolerability

**Figure 1:**
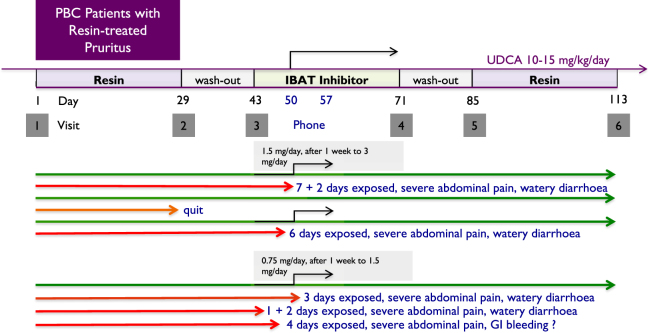
A4250PBCpruritus study flow scheme (upper) and individual outcome of the ten participating patients.

The first six patients, all females, were supposed to start at Visit 3 with a dose of 1·5 mg A4250 per day, with the option to double the dose depending on the telephone follow-up after one week (Fig. 1). At this visit one patient had dropped out by leaving Sweden. Three of the remaining five patients were able to double the dose of A4250 after one week, to continue with 3 mg/day until Visit 4, and to finish the study per protocol. The remaining two patients finished after six and seven days, respectively, with A4250 1.5 mg/day, due to diarrhoea with more than four bowel movements per day and abdominal pain that occurred 1–2 hours after ingestion of study drug. One of these two patients restarted with A4250 1.5 mg/day after a break of four days but finished after another two days due to the same symptoms.

At this point the protocol was amended and the starting dose of A4250 was lowered by 50% to 0.75 mg/day (Fig. 1). Another four patients were enrolled, three females and one male. The clinical course was similar to the first group: Whereas one patient was able to finish the study per protocol, including doubling of the dose of A4250 to 1.5 mg/day until Visit 4, the remaining three discontinued the study medication after three to four days due to abdominal pain (three) and diarrhoea (two) and melena and significantly decreased haemoglobin (one), which was registered as a severe adverse event. Upper endoscopy did not reveal a bleeding source in this patient who refused lower endoscopy since no further signs of gastrointestinal bleeding occurred during one month of follow-up. Otherwise there were no changes in routine laboratory tests in any study patient. The study was finished after nine study drug-exposed patients with five having withdrawn prematurely from study medication because of abdominal adverse events.

### Efficacy

**Figure 2:**
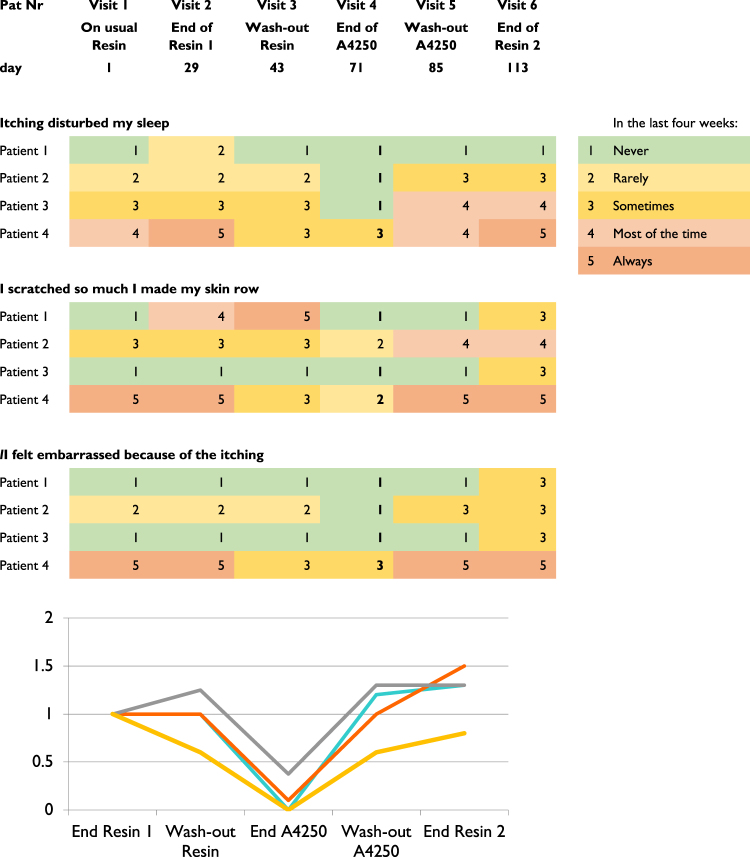
Itch intensity according to PBC-40 itch domain in the last four weeks (upper) and by Visual Analogue Score (VAS) relative to End of Resin 1 in patients that finished per protocol.

All nine patients that were exposed to study medication reported improvements of pruritus starting already on the second day of medication, both at 1.5 and 0.75 mg/day of A4250, which was the reason why two patients that had experienced abdominal side effects tried A4250 again for a couple of days. In those four patients that finished per protocol, pruritus had disappeared completely in two of them and was improved by 60% and 80%, respectively, in the other two (Fig. 2), and also in them, it then occurred less than six hours per day (Supplementary Fig. 1). The substantial improvement of pruritus in these four patients was also documented in the PBC40 itch domain (Fig. 2) and the 5-D itch scale (Supplementary Fig. 1). Most importantly, these patients reported that itch did no longer disturb their sleep at night (Fig. 2, Supplementary Fig. 1) and that the itch was not so severe anymore that it would embarrass them in their everyday activity (Supplementary Fig. 1).

Furthermore, by the end of the A4250 treatment period the itch had largely disappeared from all parts of the body according to the 5-D itch scale (Supplementary Fig. 2). Most noteworthy, two of these four patients had severe, in part bleeding scratch marks due to the itch on both lower limbs that disappeared during treatment with A4250 but returned afterwards during wash-out and treatment with bile acid sequestrants that overall was not reported to have any significant effect on pruritus.

### Exploratory metabolic investigations

The low number of patients that finished the study per protocol did not allow a formal statistical evaluation, explaining why only descriptive data relative to baseline measurements at Visit 2 are presented here. Liver enzymes (AST, ALT, ALP), bilirubin and lipid profiles (total cholesterol, HDL, LDL and triglycerides) did not change by A4250 (Supplementary Figs 3, 4). Total serum bile acid levels where 32.4 ± 10.6 μmol/L at inclusion (n = 10) with no differences between those that finished the study per protocol (25.9 ± 4.9 μmol/L, n = 4) and those that prematurely stopped taking A4250 (30.6 ± 9.9 μmol/L, n = 5). Total UDCA (sum of unconjugated and glycine- or taurine-conjugated UDCA) consisted of 61.5 ± 4.9% of total bile acids at inclusion (n = 10). There were also no consistent trends in total serum bile acid concentrations (and the relative amount of UDCA) and FGF19 levels whereas in three out of four patients that finished per protocol bile acid synthesis marker C4 increased substantially (Supplementary Fig. 5). Lysophosphatidic acid, which is a potential mediator of cholestatic pruritus, was measured by autotaxin activity that in four patients at baseline (Visit 2) was between 6.9 and 11.8 nmol/ml/min, which is in the lower range of PBC patients^14^. We found a decrease in three out of four patients, and the fourth patient had unchanged autotaxin activity values (Supplementary Fig. 5).

## Discussion

In our pilot study with the IBAT inhibitor A4250 for the treatment of pruritus in PBC we made two major observations: First, the compound substantially improved cholestatic pruritus, which was verified by three different measures of itch: VAS, PBC-40 itch domain, and 5-D itch scale. In addition, as an objective marker, we observed complete healing of itch scares in the two patients that presented with this finding. Second, five out of nine patients that were exposed to study medication discontinued after two to seven days due to abdominal pain and diarrhoea. Increased bowel movements are intrinsic to the mode of action of IBAT inhibitors as these compounds can induce bile salt malabsorption diarrhoea, an effect that with a similar compound was found to be beneficial in chronic idiopathic constipation^15,16^.

Our results are in concordance with the recent randomized placebo-control phase IIa trial with the IBAT inhibitor GSK2330672 for the treatment of cholestatic pruritus in PBC patients^17^. This drug was used at 45–90 mg twice daily for two weeks and alleviated itch as estimated by VAS, PBC-40 itch domain and 5-D itch scale. It also reduced serum bile acid and FGF19 levels and increased C4 levels, similar to our findings. Of note, 33% of the patients administered GSK2330672 reported diarrhoea and a substantial number of patients reported abdominal pain, but no subject withdrew from this study which might be attributable to the twice daily administration regimen^17^. A third IBAT inhibitor has been tested in patients with PBC and pruritus (Lopixibat, 10–20 mg once daily). Compared to placebo, no significant changes in pruritus but a higher incidence of diarrhoea and abdominal pain were reported resulting in withdrawal in 2/42 patients; serum bile acid reductions and C4 increases reached nominal significance^18^. Taken together, all clinical experience with IBAT inhibitors so far, from their mode of action still give circulating bile acids a role in the pathogenesis of cholestatic pruritus, although the bile acid effect might be indirect^3,9^.

The high incidence of abdominal side effects in our bile acid sequestrant pre-treated population indicates that the start dose of A4250 may have been too high for adult patients, at least at a single dose regiment, which should be acknowledged in further studies with this compound. It can be speculated that bile acid sequestrants that notoriously induce constipation might have triggered increased bowel sensitivity before administration of A4250. Of note, the same compound that we tested in adults with PBC and cholestatic pruritus has also been studied in an open label trial in 20 paediatric patients with pruritus-complicated cholestatic liver disease^19^. In this population with various liver diseases, including progressive familial intrahepatic cholestasis, Alagille syndrome, biliary atresia, or intrahepatic cholestasis, improvements of pruritus were observed without diarrhoea or abdominal pain as side effects^19^.

In summary, A4250 seems to be a promising compound for the treatment of cholestatic pruritus for which efficient and tolerable treatment options still are missing. The major side effects (abdominal pain and diarrhoea) are most likely related to the mode of action of IBAT inhibition, which warrants careful dose regimens (split doses) in further randomized placebo-controlled studies, in particular in those patients where bowel motility might have been affected by constipating pre-treatment.

## Materials and Methods

### Study design and participants

The primary efficacy objective was to demonstrate the efficacy of once daily dosing of A4250, 1.5–3 mg orally during a four-week treatment period to relieve patients with PBC from cholestatic pruritus as determined by evaluation of effects on pruritus using the visual analogue scale (VAS), PBC-40 itch domain^20^, and the 5-D itch scale^21^. The primary safety objective of this study was to assess the safety and tolerability of A4250, 1.5–3 mg orally during a four-week treatment period, in patients with PBC and cholestatic pruritus, as determined by the occurrence of treatment-emergent SAEs. Secondary efficacy objectives of this study were to demonstrate the efficacy of A4250 orally on other pruritus variables and quality of life. Secondary safety objectives of this study included assessment of the safety and tolerability of A4250 during a four-week treatment period, as determined by the occurrence of treatment-emergent AEs and changes in other safety parameters including liver and kidney function tests and vital signs. Exploratory efficacy objectives of this study included assessment of pharmacodynamic parameters of bile acid metabolism such as bile acids, bile acid synthesis marker 7α-hydroxy-4-cholesten-3-one (C4) and fibroblast growth factor (FGF) 19 in plasma and lysophosphatidic acid formation in serum, and assessment of surrogate markers of cholestatic liver disease such as serum alkaline phosphatase, transaminases and total bilirubin.

For all participants, an approval to use A450 was provided by the Regional Ethical Review Board in Gothenburg (Dnr 795-14) and the study took place under the supervision of the Swedish Medical Products Agency (EudraCT 2014-004070-42). The pilot study was entirely investigator-initiated and performed in accordance with the protocol, good clinical practice, and all relevant guidelines and regulations. An informed consent was obtained from all participants prior to inclusion in the study. Support in the form of study medication A4250 (0.75 mg and 1.5 mg capsules) was provided by Albireo AB, Gothenburg, Sweden.

Inclusion criteria were diagnosis of PBC or PBC-autoimmune hepatitis overlap as established according to AASLD/EASL definitions. Patients had to be classified as UDCA non-responders defined as >6 months of UDCA and with serum ALP >1.67 ULN at the time of enrolment and having been on treatment with bile acid sequestrant cholestyramine at a dose >4 g BID or colestipol ≥5 g BID for at least three months, with a VAS-itch of at least 30/100 mm during the day before baseline (Visit 2). Women of childbearing capacity were allowed to participate provided a negative serum pregnancy test upon inclusion in the trial and the use of highly effective birth control or abstinence during the study.

Exclusion criteria were other liver diseases than PBC and other reasons for itching such as atopic dermatitis or other primary skin diseases, jaundice of extrahepatic origin, cancer, chronic kidney disease, and chronic severe infection.

A flow scheme of the study is shown in Fig. 1. From Visit 1 (Baseline) patients were instructed to strictly adhere to the previous treatment with bile acid sequestrant for four weeks until Visit 2. After a two-week wash out at Visit 3, 1.5 mg (n = 5) and 0.75 mg (n = 4) capsules, respectively, were started with the option to double the dose according to a telephone consultation after one week. Treatment with A4250 was intended for a total of four weeks until Visit 4, followed by a two-week wash-out (Visit 5) and return to previous bile acid sequestrant treatment four another four weeks (Visit 6).

Through the entire study, patients were instructed to use a paper diary daily to indicate medications, number of bowel movements, stool consistency according to the Bristol Stool Chart^22^, and abdominal pain (0 = none, 1 = moderate, 2 = severe). At each visit in addition to vital parameters, severity of pruritus during the last two weeks was assessed as (i) visual analogue score (VAS, 0–100 mm scale), (ii) the PBC-40 itch domain^20^, and (iii) by the 5-D itch scale^21^.

### Biochemical analysis

Blood was drawn from patients for biochemical analysis at all visits and serum liver tests (AST, ALT, ALP, Bilirubin) and lipids (serum total cholesterol, HDL-cholesterol, LDL-cholesterol, triglycerides) were analysed by routine clinical chemistry. Plasma bile acid and C4 levels were quantified by ultra-performance liquid chromatography-tandem mass spectrometry (UPLC-MS/MS) and compared to unlabelled and deuterium-labelled reference bile acids^23^. Plasma FGF19 was measured by ELISA according to the manufacturer’s handbook (R&D Systems, Minneapolis USA). Serum autotaxin activity was measured by a previously published assay^24^.

### Statistical analysis

Because of the low number of participants and the fact that this was an exploratory study, no formal statistical evaluation was applicable.

### Data availability statement

The datasets generated during and/or analysed during the current study are available from the corresponding author on reasonable request.

## Electronic supplementary material

Supplementary Information
