# Supplementary material for: Pilot study with IBAT inhibitor A4250 for the treatment of cholestatic pruritus in primary biliary cholangitis
Source: Sci Rep. 2018 Apr 27;8:6658. doi: 10.1038/s41598-018-25214-0 (PMC5923243; doi:10.1038/s41598-018-25214-0)
Supplement: Supplementary file 1 — Supplementary Information [file 41598_2018_25214_MOESM1_ESM.doc]

**Pilot study with IBAT inhibitor A4250 for the treatment of cholestatic pruritus in primary biliary cholangitis.**

^1^Samer Al-Dury, ^1^Annika Wahlström, ^2^Staffan Wahlin, ^3^Jacqueline Langedijk, ^3^Ronald Oude Elferink, ^1^Marcus Ståhlman, and ^1^Hanns-Ulrich Marschall

^1^Sahlgrenska Academy, Institute of Medicine, Department of Molecular and Clinical Medicine and Wallenberg Laboratory, University of Gothenburg, Gothenburg, ^2^Karolinska University Hospital Huddinge, Department of Gastroenterology and Hepatology, Stockholm, Sweden,

^3^Tytgat Institute for Liver and Intestinal Research, Department of Gastroenterology and Hepatology, Academic Medical Center, University of Amsterdam, Amsterdam, The Netherlands

**Corresponding author:**

Hanns-Ulrich Marschall, M.D., Ph.D.

Department of Molecular and Clinical Medicine

Institute of Medicine, Sahlgrenska Academy

University of Gothenburg

S-41345 Gothenburg, Sweden

Phone: +46 70 8774073

Fax: +46 31 34827458

E-mail: [hanns-ulrich.marschall@gu.se](mailto:hanns-ulrich.marschall@gu.se)

**Supplementary Information**

| ***Q1 - DURATION - During the last 2 weeks, how many hours a day have you been itching?*** | | | | | | | | | | | | | | | |  | | | | |
| --- | --- | --- | --- | --- | --- | --- | --- | --- | --- | --- | --- | --- | --- | --- | --- | --- | --- | --- | --- | --- |
|  |  |  |  |  |  |  |  |  | |  | | | | | | |  | | | |
|  |  |  |  |  |  |  |  |  | |  | | | | | | |  | | | |
| **Pat Nr** | **V1** | **V2** | **V3** | **V4** | **V5** | **V6** |  |  | |  | | | | | | |  | | | |
| Patient 1 | 1 | 1 | 2 | **1** | 1 | 1 |  | 1 | | < 6 hr | | | | | | |  | | | |
| Patient 2 | 1 | 1 | 1 | **1** | 1 | 3 |  | 2 | | 6-12 hr | | | | | | |  | | | |
| Patient 3 | 2 | 3 | 1 | **1** | 2 | 4 |  | 3 | | 12 -18 hr | | | | | | |  | | | |
| Patient 4 | 5 | 5 | 1 | **1** | 2 | 5 |  | 4 | | 18-23 hr | | | | | | |  | | | |
|  |  |  |  |  |  |  |  | 5 | | whole day | | | | | | |  | | | |
|  | | | | | | | | |  | |  | | | | | |  | | | |
| ***Q2 - DEGREE - Please rate the intensity of your itching over the past 2 weeks*** | | | | | | | | | | | |  | |  | | | | |  | |
|  |  |  |  |  |  |  |  |  | |  | | | | | | |  | | | |
| **Pat Nr** | **V1** | **V2** | **V3** | **V4** | **V5** | **V6** |  |  | |  | | | | | | |  | | | |
| Patient 1 | 2 | 3 | 4 | **1** | 2 | 2 |  | 1 | | no itch | | | | | | |  | | | |
| Patient 2 | 3 | 3 | 2 | **3** | 3 | 4 |  | 2 | | mild itch | | | | | | |  | | | |
| Patient 3 | 2 | 3 | 2 | **2** | 3 | 4 |  | 3 | | moderate itch | | | | | | |  | | | |
| Patient 4 | 5 | 5 | 1 | **2** | 3 | 5 |  | 4 | | severe itch | | | | | | |  | | | |
|  |  |  |  |  |  |  |  | 5 | | unbearable itch | | | | | | |  | | | |
|  |  |  |  |  |  |  |  |  | |  | | | | | | |  | | | |
|  | | | | | | | | | | | | | | | | |  | | | |
| ***Q3 - DIRECTION - Over the past 2 weeks has your itching gotten better or worse compared to the previous month?*** | | | | | | | | | | | | | | | | |  | | | |
|  |  |  |  |  |  |  |  |  | |  | | | | | | |  | | | |
| **Pat Nr** | **V1** | **V2** | **V3** | **V4** | **V5** | **V6** |  |  | |  | | | | | | |  | | | |
| Patient 1 | 4 | 5 | 4 | **1** | 5 | 3 |  | 1 | | gone | | | | | | |  | | | |
| Patient 2 | 4 | 4 | 2 | **3** | 4 | 5 |  | 2 | | much better | | | | | | |  | | | |
| Patient 3 | 4 | 5 | 2 | **3** | 5 | 5 |  | 3 | | little better | | | | | | |  | | | |
| Patient 4 | 5 | 5 | 3 | **2** | 5 | 5 |  | 4 | | unchanged | | | | | | |  | | | |
|  |  |  |  |  |  |  |  | 5 | | worsened | | | | | | |  | | | |
|  |  |  |  |  |  |  |  |  | |  | | | | | | |  | | | |
|  | | | | | | | | | | | | |  | | | | |  | | |
| ***Q4 - DISABILITY - Rate the impact of your itch on the following activities over the last 2 weeks*** | | | | | | | | | | | | | | |  | | | | |  |
|  |  |  |  |  |  |  |  |  | |  | | | | | | |  | | | |
| **Pat Nr** | **V1** | **V2** | **V3** | **V4** | **V5** | **V6** |  |  | |  | | | | | | |  | | | |
|  | **Sleep** | | | | | |  |  | |  | | | | | | |  | | | |
| Patient 1 | 1 | 2 | 2 | **1** | 2 | 2 |  | 1 | | Never effects sleep | | | | | | |  | | | |
| Patient 2 | 2 | 2 | 1 | **2** | 2 | 1 |  | 2 | | Occasionally delays falling asleep | | | | | | |  | | | |
| Patient 3 | 2 | 3 | 2 | **1** | 4 | 5 |  | 3 | | Frequently delays falling asleep | | | | | | |  | | | |
| Patient 4 | 5 | 5 | 2 | **1** | 3 | 4 |  | 4 | | Delays falling asleep and occasionally wakes me up at night | | | | | | |  | | | |
|  |  |  |  |  |  |  |  | 5 | | Delays falling asleep and frequently wakes me up at night | | | | | | |  | | | |
|  |  | | | | | |  |  | |  | | | | | | |  | | | |
|  | **Leisure / Social** | | | | | |  | 0 | | N/A | | | | | | |  | | | |
| Patient 1 | 1 | 1 | 1 | **0** | 1 | 1 |  | 1 | | Never affects this activity | | | | | | |  | | | |
| Patient 2 | 1 | 1 | 0 | **0** | 0 | 1 |  | 2 | | Rarely affects this activity | | | | | | |  | | | |
| Patient 3 | 1 | 1 | 1 | **1** | 1 | 3 |  | 3 | | Occasionally affets this activity | | | | | | |  | | | |
| Patient 4 | 3 | 3 | 2 | **1** | 1 | 3 |  | 4 | | Frequently affects this activity | | | | | | |  | | | |
|  |  |  |  |  |  |  |  | 5 | | Always affetcs this activity | | | | | | |  | | | |
|  |  | | | | | |  |  | |  | | | | | | |  | | | |
|  | **Housework / Errands** | | | | | |  | 0 | | N/A | | | | | | |  | | | |
| Patient 1 | 1 | 1 | 1 | **0** | 1 | 1 |  | 1 | | Never affects this activity | | | | | | |  | | | |
| Patient 2 | 1 | 1 | 0 | **0** | 0 | 1 |  | 2 | | Rarely affects this activity | | | | | | |  | | | |
| Patient 3 | 1 | 1 | 1 | **1** | 1 | 3 |  | 3 | | Occasionally affets this activity | | | | | | |  | | | |
| Patient 4 | 3 | 3 | 2 | **1** | 1 | 3 |  | 4 | | Frequently affects this activity | | | | | | |  | | | |
|  |  |  |  |  |  |  |  | 5 | | Always affects this activity | | | | | | |  | | | |
|  |  |  |  |  |  |  |  |  | |  | | | | | | |  | | | |
|  |  | | | | | |  |  | |  | | | | | | |  | | | |
|  | **Work / School** | | | | | |  | 0 | | N/A | | | | | | |  | | | |
| Patient 1 | 1 | 1 | 1 | **0** | 1 | 1 |  | 1 | | Never affects this activity | | | | | | |  | | | |
| Patient 2 | 1 | 1 | 0 | **0** | 0 | 1 |  | 2 | | Rarely affects this activity | | | | | | |  | | | |
| Patient 3 | 1 | 1 | 1 | **1** | 1 | 1 |  | 3 | | Occasionally affects this activity | | | | | | |  | | | |
| Patient 4 | 0 | 1 | 1 | **0** | 0 | 0 |  | 4 | | Frequently affects this activity | | | | | | |  | | | |
|  |  |  |  |  |  |  |  | 5 | | Always affetcs this activity | | | | | | |  | | | |

**Supplementary Figure 1:** Description of type and intensity of pruritus according to 5-D itch scale questionnaire in patients that finished the study per protocol.


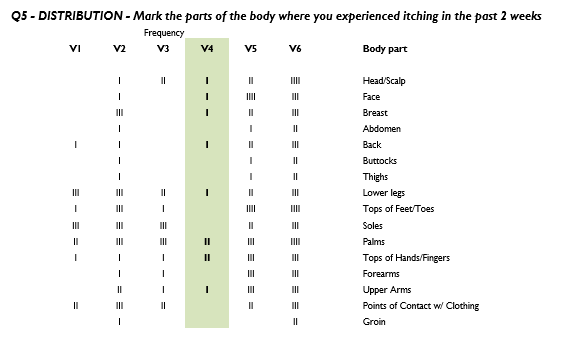


**Supplementary Figure 2:** Description of distribution of pruritus according to 5-D itch scale questionnaire in patients that finished the study per protocol.


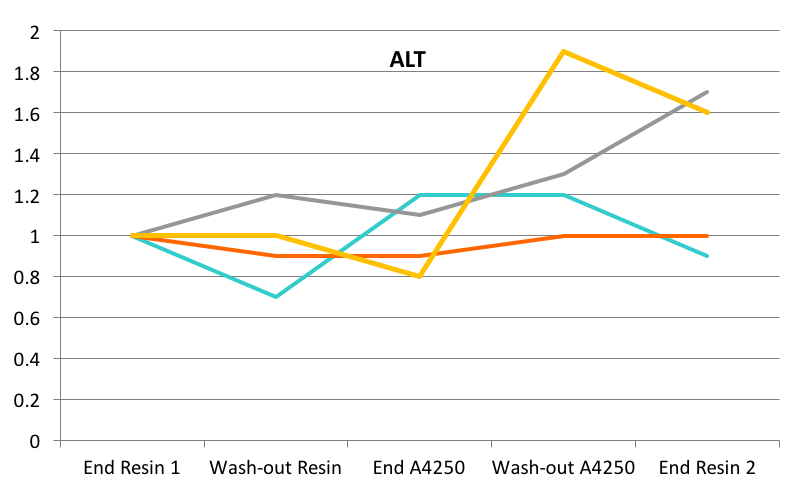

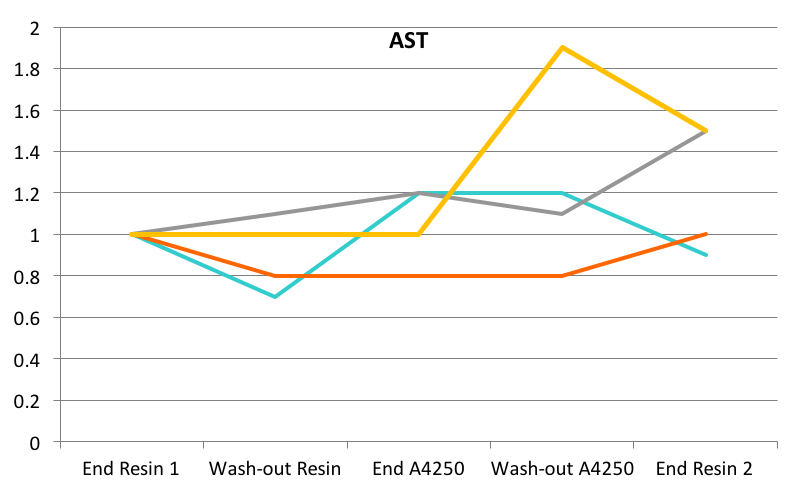


**Supplementary Figure 3A:** Relative changes of liver enzymes (ALT, AST) in patients that finished per protocol, compared to End of Resin 1.


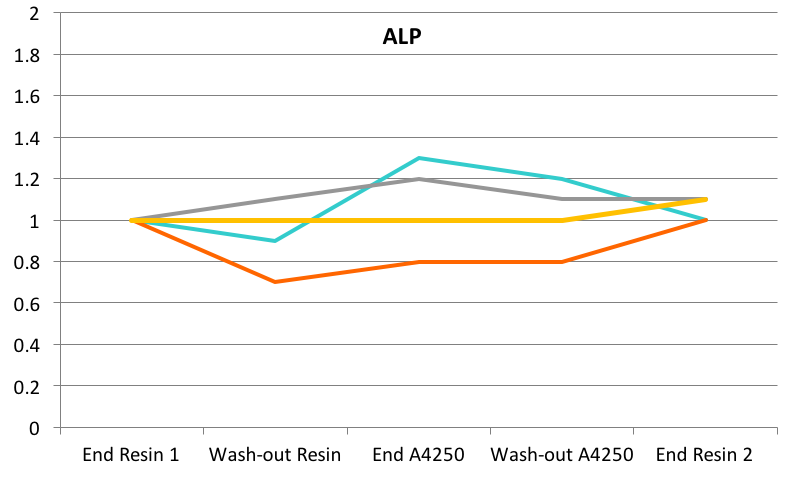

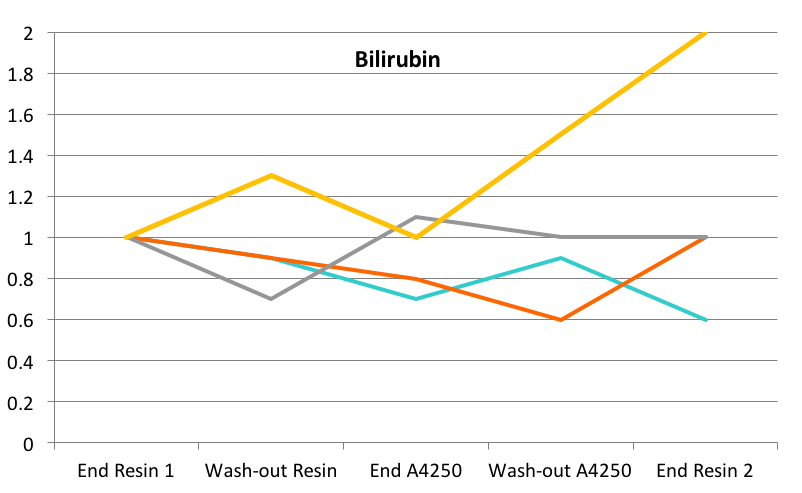


**Supplementary Figure 3B:** Relative changes of liver enzymes (ALP and bilirubin) in patients that finished per protocol, compared to End of Resin 1.


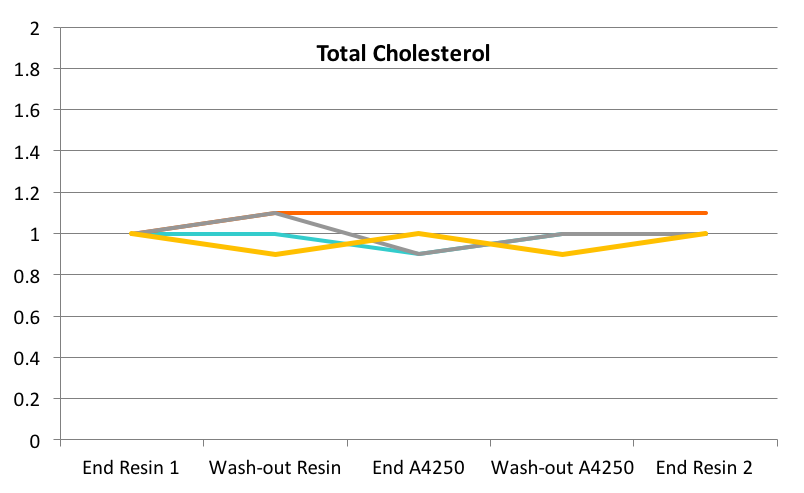


**‘**
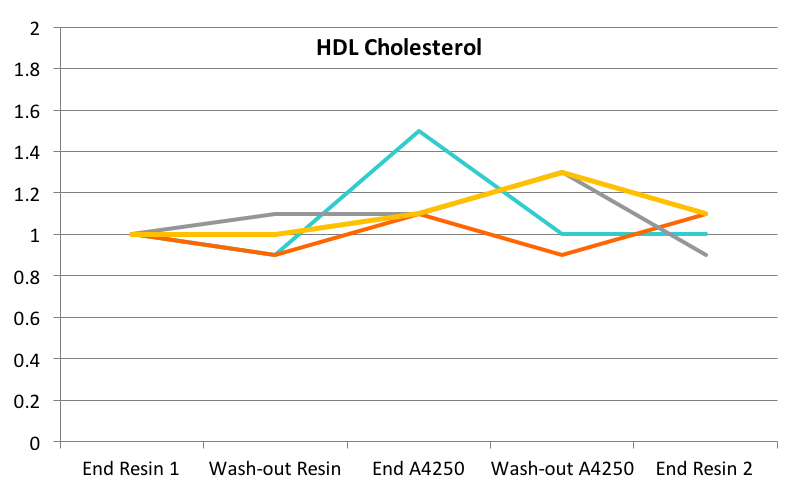


**Supplementary Figure 4A:** Relative changes of serum total cholesterol and HDL-cholesterol in patients that finished per protocol, compared to End of Resin 1.


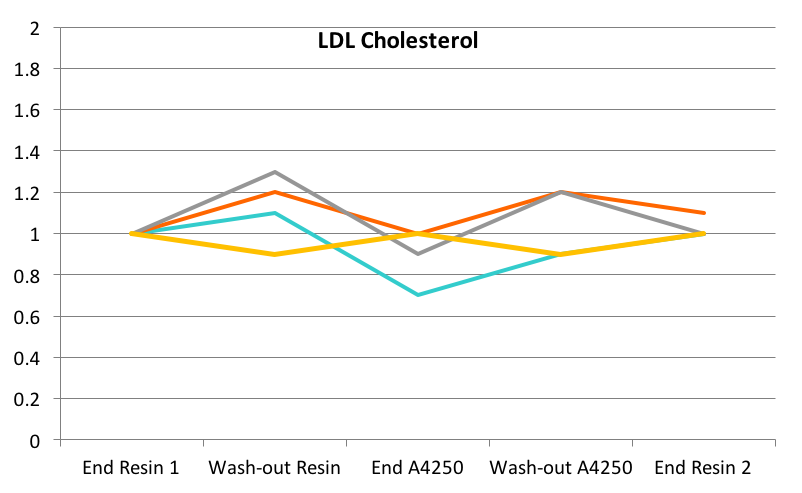


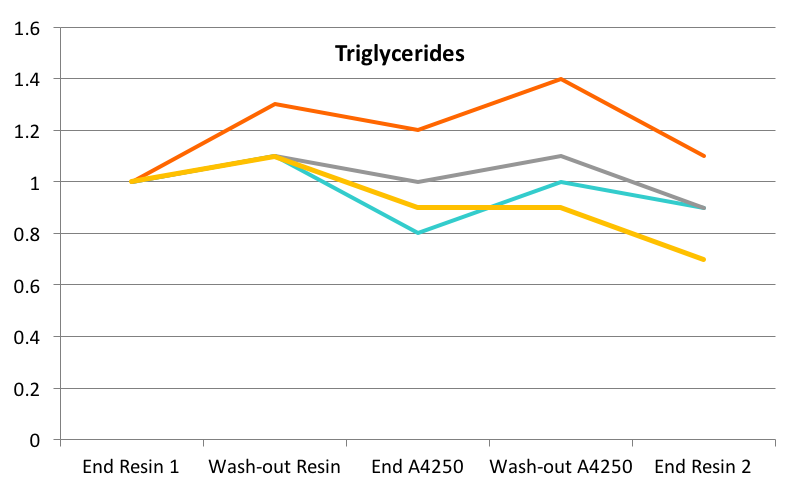


**Supplementary Figure 4B:** Relative changes of serum LDL-cholesterol, and triglycerides in patients that finished per protocol, compared to End of Resin 1.


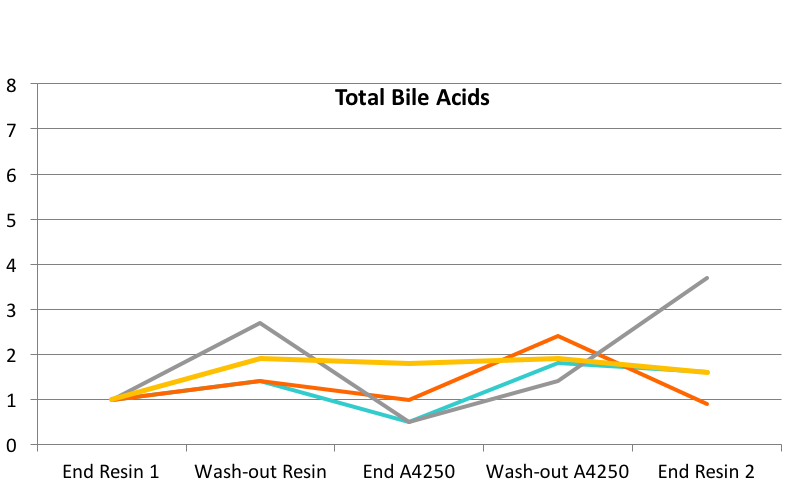


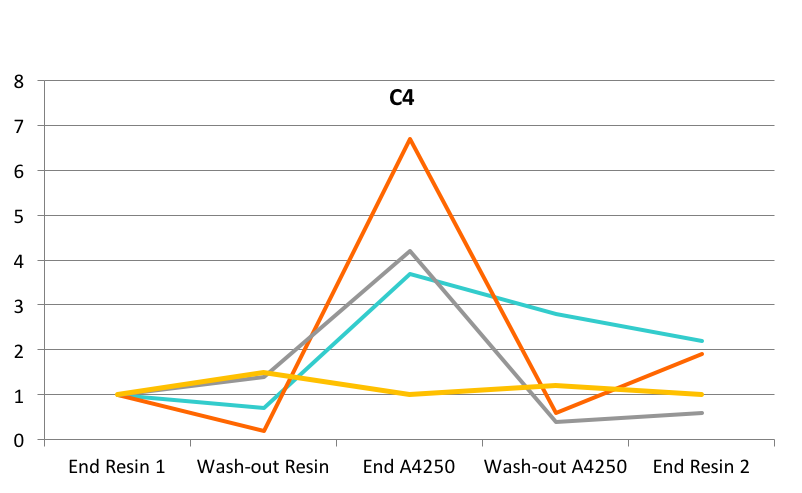


**Supplementary Figure 5A:** Relative changes in total serum bile acids and bile acid synthesis marker 7α-hydroxy-4-cholesten-3-one (C4) in patients that finished per protocol, compared to End of Resin 1.


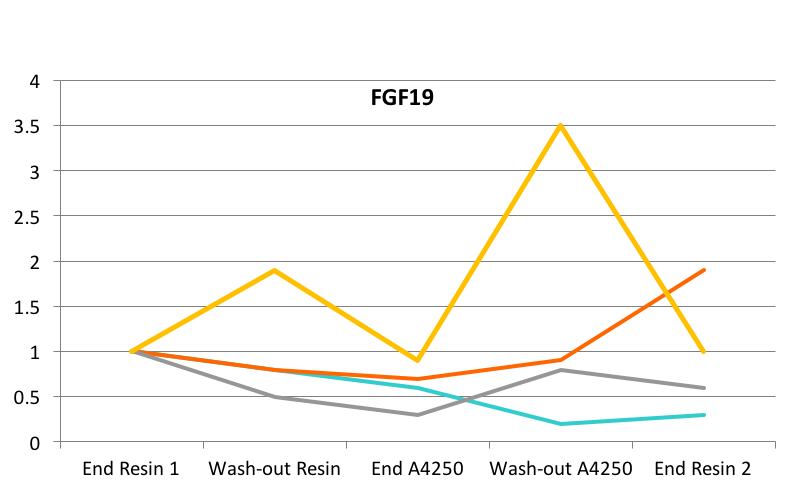


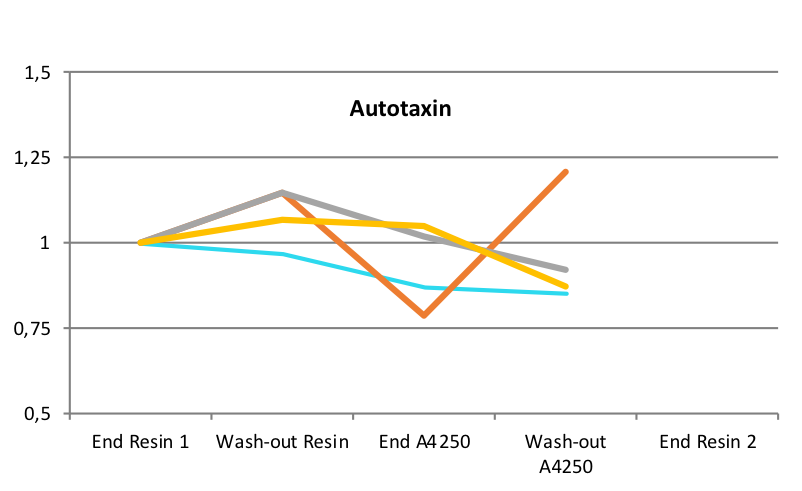


**Supplementary Figure 5B:** Relative changes in total circulating FGF19 and autotaxin activity in patients that finished per protocol, compared to End of Resin 1.
